# Adsorption of Direct Blue 78 Using Chitosan and Cyclodextrins as Adsorbents

**DOI:** 10.3390/polym11061003

**Published:** 2019-06-05

**Authors:** Ainoa Murcia-Salvador, José A. Pellicer, María I. Fortea, Vicente M. Gómez-López, María I. Rodríguez-López, Estrella Núñez-Delicado, José A. Gabaldón

**Affiliations:** 1 Health Sciences Ph.D. Program, Universidad Católica de Murcia (UCAM), Campus de los Jerónimos, 135, Guadalupe, 30107 Murcia, Spain; amurcia6@alu.ucam.edu; 2 Dpto. de Ciencias de la Salud, Universidad Católica de Murcia (UCAM), Campus de los Jerónimos, 135, Guadalupe, 30107 Murcia, Spain; japellicer@ucam.edu (J.A.P.); mifortea@ucam.edu (M.I.F.); vmgomez@ucam.edu (V.M.G.-L.); mirodriguez@ucam.edu (M.I.R.-L.); enunez@ucam.edu (E.N.-D.)

**Keywords:** adsorption, chitosan, β-CDs, Direct Blue, kinetics, isotherm, pulsed light

## Abstract

The dyeing industry is one of the most polluting in the world. The adsorption of dyes by polymeric matrixes can be used to minimize the discharge of dyes into the environment. In the present study, chitosan-NaOH and β-cyclodextrin-epichlorohydrin polymers were used to remove the dye Direct Blue 78 from a wastewater model. To understand the adsorption behavior of Direct Blue 78 onto the polymers, adsorption rate and maximum adsorption capacity were calculated using kinetic tests and isotherm curves respectively. The kinetic data and mechanism of the adsorption process were analyzed by three models and the equilibrium data by three adsorption isotherms; also the different thermodynamic parameters were calculated. Results showed that the adsorption process follows pseudo-second-order kinetics in both polymers and the Langmuir isotherm best-fitted data for chitosan-NaOH polymer and the Freundlich isotherm for the β-CDs-EPI polymer. The adsorption process is exothermic in both cases and spontaneous for the β-CDs-EPI polymer to a certain temperature and not spontaneous for the chitosan-NaOH polymer and β-CDs-EPI polymer at higher temperatures. The complementary action of an advanced oxidation process eliminated >99% of the dye from water. The coupled process seems to be suitable for reducing the environmental impact of the dyeing industry.

## 1. Introduction

Currently, many industries generate a considerable amount of polluted wastewater because of the consumption of large amounts of water and the employment of chemicals during manufacturing and dyeing of their products [1]. 

Dyes have been widely used for thousands of years for textile, paint and other applications because of their favorable characteristics, such as bright color, simple application and water-fastness [2]. Today, over 100,000 commercially available different dye types exist and more than 7 × 10^5^ tons are generated annually [3].

Dyes are non-biodegradable, stable, resistant to light, heat, oxidizing agents and potentially carcinogenic and toxic, even at small concentrations; therefore, their release into the environment poses serious ecological, aesthetical and health problems (skin irritations, dysfunction of the kidney, respiratory problems and increase cancer risk in humans) [4]. Moreover, their discharge into the environment may negatively affect photosynthetic processes of aquatic plants, reducing oxygen levels in water and, in serious cases, resulting in the suffocation of aquatic fauna and flora [5]. 

Commercial dyes are usually categorized based on their color, functional groups, chemical structure and application. Furthermore, dyes are classified into cationic, anionic and nonionic according to the produced particle charge upon dissolution in an aqueous medium. Due to the toxicity of amine groups in azo dyes, they are considered very dangerous for human health and the environmental life, consequently, dye industries have been pressed to remove dyes from their wastewater effectively to ensure safe discharge into the environment [6]. 

In order to remove dye from wastewater and to improve the quality of treated wastewater discharged into the environment, it is necessary to select a correct treatment method. As a result, different methods have been applied for the effective treatment of dye wastewater [7]. Several conventional methods such as adsorption on activated carbons, coagulation and flocculation, chemical oxidation, reverse osmosis, bacterial action, activated sludge, ozonation, membrane filtration, ion exchange and electrochemical techniques are the commonly used methods for the removal of dyes from wastewater [8]. 

These methods may be able to reduce the color of the wastewater but they exhibit several limitations such as high cost, low effectiveness and generation of excess sludge so that some are inappropriate for use by small-scale industries [7]. Among them, adsorption is considered the most economical method [9]. Adsorption, which is a rapid method and convenient for toxic contaminants, it is one of the easiest and effective physio-chemical treatment processes for dye removal, with low initial costs, producing nontoxic by-products, flexibility and simplicity of design, fast adsorption rate, facile separation and simple and user-friendly. Recently, materials based on natural polymers have been developed [10]. 

Cyclodextrins (CDs) are cyclic oligosaccharides obtained from the enzymatic degradation of starch and they are used widely in separation science because CD-complexation phenomena serve for separation of compounds and extraction processes, and provide a versatile and useful tool for protecting the environment [11]; however, over the last several decades, biodegradable and eco-friendly polymers have been developed as an alternative to epichlorohydrin polymers demanding less chemical treatment during production [12].

Chitosan is used as an alternative adsorbent with a demonstrated adsorption capacity to conventional wastewater treatment processes [10]. The use of biopolymers such as chitin and chitosan is one of the new adsorption methods for the removal of heavy metal ions and dyes [8]. Chitin is an important crustacean by-product produced by the shellfish processing industry that reduces the waste and its negative impact on the environment [13]. Chitosan is a natural biopolymer synthesized from the deacetylation of chitin, which is the second most abundant polysaccharide in nature, consisting mainly of unbranched chains of β-(1-4)-2-acetoamido-2-deoxy-d-glucose, and it is obtained from crustacean shells such as crabs, crayfish, lobster prawns, fungi, insects and other crustaceans [14]. Chitosan is a heterogeneous, linear, cationic polysaccharide with a high molecular weight and hydrophobic that possesses multiple properties such as biodegradability, hydrophilicity, non-toxicity, biocompatibility or adsorption properties [8].

Since the retention of dyes by polymers is not 100% efficient, unadsorbed amounts of dyes still pose an ecological issue and a complementary method is required in order to minimize the amount of dye that will be eventually disposed to the environment. Advanced oxidation processes (AOPs) can be used for this purpose. AOPs are a group of methods based on the generation of the highly oxidant hydroxyl radicals, which oxidize organic molecules. AOPs based on pulsed light (PL) uses high-intensity short-time pulses of light of wide-spectrum rich in UV wavelengths to generate hydroxyl radicals from hydrogen peroxide. It has been tested for the degradation of textile dyes successfully [15].

In the present study, chitosan-NaOH polymer beads and β-CDs-EPI polymers were prepared in order to analyze the adsorption efficiency of both adsorbents using Direct Blue 78 dye (DB78) due to the high amount used in the textile industry and its high aqueous solubility. These properties make DB78 a suitable dye to study their adsorption properties. To achieve this goal, the experimental data were fitted to different models and isotherms to elucidate the adsorption characteristics of each one. To remove the remaining dye in the aqueous solution after treatment, an advanced oxidation process was considered.

## 2. Materials and Methods 

### 2.1. Chemicals

Chitosan powder, sodium hydroxide, acetic acid, sodium borohydride, acetone and epichlorohydrin were obtained from Sigma-Aldrich (Madrid, Spain), β-CDs were from AraChem (Tilburg, The Netherlands). Direct Blue 78 was provided by Colorprint (Alcoy, Spain).

### 2.2. Chitosan-NaOH Polymer Preparation

Chitosan (CAS 9012-76-4) was prepared by dissolving 12 g of chitosan powder in a 600 mL 2% (*v*/*v*) acetic acid aqueous solution. The homogenous gel was obtained by continual stirring 4 h at room temperature. To obtain polymer beads, the gelled solution was forced through a micropipette. The solution was dropped into a bath containing a NaOH aqueous solution and spherical particles quickly formed [12].

### 2.3. β-CDs-EPI Polymer Preparation

The β-CDs-EPI polymer was prepared using the following method: 30 mg of sodium borohydride were mixed with 12 g of CDs, in 12 mL of water. After stirring for 10 min at 50 °C, 13 mL of sodium hydroxide (40%) was added to the solution and this was stirred 5 min and then 132 g of EPI were added dropwise. This mixture was stirred for 6 h at 50 °C until total polymerization. The polymer was washed with acetone for 10 min. Finally, the polymer was dried in an oven overnight at 50 °C, with the purpose of removing the residual acetone and thoroughly dry the polymer [16]. 

### 2.4. Dye Solution Preparation

**Figure 1:**
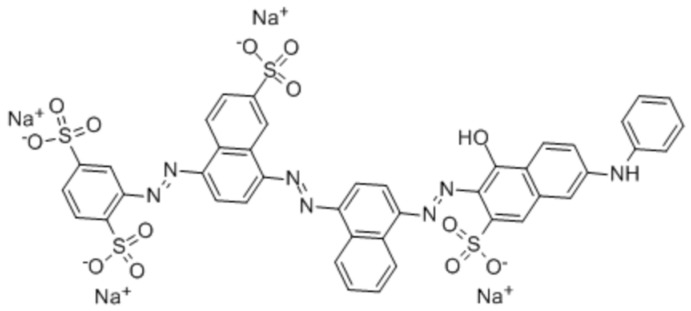
The molecular structure of DB78.

The adsorption capacity out of the polymers was tested using Direct Blue 78 dye as a model compound. DB78 is an azo dye (CAS 2503-73-3) whose molecular weight is 1055.91 g/mol and its formula is C_42_H_25_N_7_Na_4_O_13_S_4_ (Figure 1). Dye concentrations of 25, 50, 100, 150, 200, 250 and 300 mg/L were used to evaluate the adsorption capacity of the different polymers.

### 2.5. Analyses and Data Evaluation

**Figure 2:**
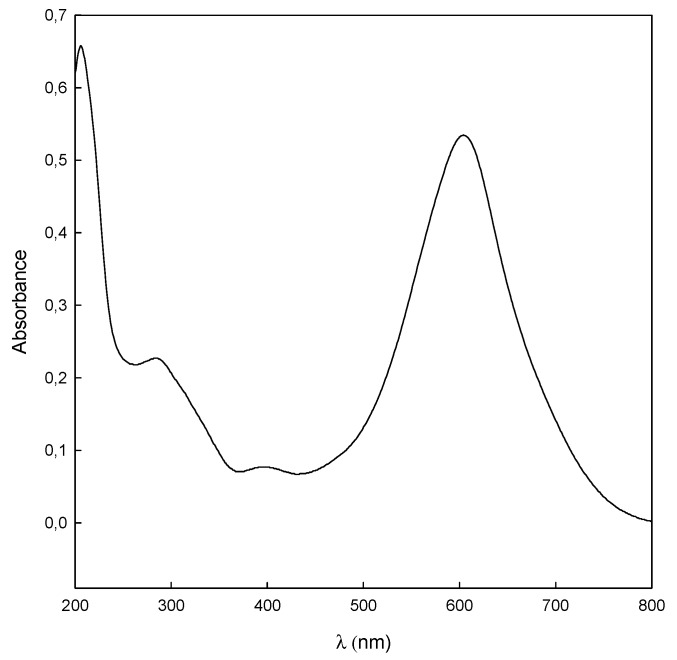
The UV-Vis spectrum of DB78 (25 mg/L) in aqueous solution.

The concentration of dye was analyzed at the maximum absorbance of the dye, 612 nm (Figure 2), using a spectrophotometer (Shimadzu UV-1603, Shimadzu Europe GmbH, Duisburg, Germany), measuring the absorbance before and after the treatment.

### 2.6. Polymer Characterization

#### 2.6.1. Porosity

The porosity and apparent density of the polymers were calculated using the following equations:

(1) Porosity (%)=Vt−VaVt∗100=Vt−MaρVt∗100

(2) $$Density\;\left(\rho \right)=\frac{V_{t}}{M_{a}}$$

where *V_t_* (cm^3^) is the total volume of polymers, *V_a_* (cm^3^) is the actual volume of the material, *M_a_* (g) is the mass of the polymers and *ρ* (g/cm^3^) is the density of the material. The experiments were repeated three times [17].

#### 2.6.2. Swelling Capacity 

The swelling capacity of the polymers was measured by using a gravimetric method: 1 g of dry samples was immersed in 200 mL of distilled water at room temperature for 3 h to reach swelling equilibrium. After this time, both polymers were filtered to remove unabsorbed water. The swelling equilibrium (*Q_eq_*, *g*/*g*) was determined according to the following equation:

(3) $$Q_{eq}=\frac{W_{s}-W_{d}}{W_{d}}$$

where *W_s_* is the mass of swollen polymers (g) and *W_d_* is the mass of dried polymers (g). The experiments were repeated three times [18].

#### 2.6.3. Particle Size Distribution

The particle size distribution was measured using a laser light diffraction instrument, Mastersizer 3000 (Malvern Instruments, Malvern, UK). The particle size was expressed as the mean volumetric size D_[4:3]_ (De Brouckere mean diameter), which is the mean diameter of a sphere with the same volume and is generally used to characterize a particle.

### 2.7. Adsorption Experiments

The adsorption experiments were carried out at 25 °C using different dye concentrations, ranging from 25 to 300 mg/L. In each experiment, 1 g of polymer was added to 40 mL of the solution of dye. The mixture was agitated at a fixed speed of 500 rpm. The remaining concentration of dye in the solution was measured every 10 min until equilibrium was reached and the maximum adsorption capacity was obtained with 1 g of adsorbent. The solid phase was separated by centrifugation at 3000 rpm for 5 min to remove all impurities that could affect the following measurement.

The adsorption capacity of dye on the polymers (*q_e_*), in mg/g, was determined by the following Equation (4) [6]:

(4) $$q_{e}=\frac{V\;\left(C_{o}-C_{e}\right)}{m}$$

where *C_o_* and *C_e_* are the initial and equilibrium values of dye concentrations in the liquid phase (mg/L), respectively, *m* is the weight of the polymer used (g) and *V* is the volume of dye solution (L). The experiments were carried out in triplicate under identical conditions. The mechanism of DB78 adsorption was explained with three well-known isotherms, adsorption kinetics and the thermodynamic study.

### 2.8. Adsorption Kinetics

With the objective to investigate the mechanism of dye adsorption onto β-CDs-EPI and chitosan-NaOH polymers, three kinetic models could be considered in order to evaluate the adsorption processes: pseudo-first-order kinetic model [19], pseudo-second-order kinetic model [20] and intraparticle diffusion model [21].

The linear form of Lagergren’s equation for first-order kinetics is given by Equation (5) [22]:

(5) $$\log\left(q_{e}-q_{t}\right)=\log q_{e}-\frac{k_{1}}{2.303}$$

where *q_t_* and *q_e_* are the amounts of dye adsorbed (mg/g) at time *t* (min) and at equilibrium respectively and *k_1_* (min^−1^) is the pseudo-first-order rate constant for the adsorption process. Values of *k_1_* were determined from the plot of ln (*q_e_* − *q_t_*) versus *t*.

If the adsorption process is a pseudo-second-order model, the linear form of the Ho and McKay rate equation is described as follows (6):

(6) $$\frac{t}{q_{t}}=\frac{1}{k_{2}q_{e}{}^{2}}+\frac{1}{q_{e}}t$$

where *q_e_* and *q_t_* are the amounts of dye adsorbed (mg/g) at equilibrium and at time *t* (min), respectively, and *k_2_* (g/mg min) is the pseudo-second-order rate constant and its value can be obtained experimentally from the intercept and slope of plot *t/q_t_* versus *t* [20].

In order to know the rate determining step in the liquid adsorption systems, the kinetic results were also analyzed by the intra-particle diffusion model to explain the adsorption behavior of DB78 on both polymers. The dye adsorption may be governed generally by either the liquid phase mass transport rate or the intraparticle mass transport rate. The rate-limiting step may be either the boundary layer (film) or the intraparticle diffusion (pore) of the solute on the solid surface from the bulk of the solution in a batch process. In diffusion studies, the rate can be expressed in terms of the square root time. According to the intraparticle diffusion model proposed by Weber and Morris [21], the root time dependence can be determined from Equation (7):

(7) $$q_{t}=k_{i}\sqrt{t}+C$$

where *q_t_* is the amount of solute on the surface of the sorbent at time *t* (mg/g), *k_i_* is the rate constant of the intraparticle diffusion model (mg/g min^1/2^), *t* the time and *C* is the intercept (mg/g). The *k_i_* values can be determined from the slopes of plots *q_t_* versus *t*^1/2^.

### 2.9. Isotherm Analysis

The adsorption equilibrium provides fundamental physicochemical data for evaluating the applicability of adsorption processes. Equilibrium isotherm equations are used to describe experimental sorption data [23]. The adsorption isotherms describe how pollutants interact with adsorbent materials and so, are important in optimizing the adsorption conditions of the analyzed polymers [24]. There are different available isotherms, in this study, Freundlich, Langmuir and Temkin isotherms were fitted to the experimental data.

Freundlich isotherm model is applicable for adsorption on heterogeneous surfaces and multilayer sorption. The use of this model suggests that adsorption energy exponentially decreases on the completion of the sorption centers of an adsorbent. This isotherm is employed to describe heterogeneous systems and is obtained by the linear form of the Freundlich expression (8) [25]:

(8) $$ln\;q_{e}=\ln K_{F}+\frac{1}{nF}\ln C_{e}$$

where *q_e_* is the amount of dye adsorbed at equilibrium time (mg/g), *C_e_* is equilibrium concentration dye in solution (mg/L), *K_F_* the Freundlich constant (L/g) related to the bonding energy and 1/*n_F_* is the heterogeneity factor. The values of 1/*n_F_* and *K_F_* are obtained from the slope and intercept of plots ln *q_e_* versus ln *C_e_*, respectively.

The Langmuir isotherm model considers that the adsorption process happens onto a surface containing a finite number of identical sites. This model is probably the most known adsorption isotherm and is extensively used for the adsorption of a pollutant from a liquid solution. The linearized form of the model is given by Equation (9) [22,26]:

(9) $$\frac{C_{e}}{q_{e}}=\frac{1}{K_{L}}+\frac{a_{L}}{K_{L}}C_{e}$$

where *C_e_* (mg/L) and *q_e_* (mg/g) are the liquid phase concentration and solid phase concentration of dye at equilibrium respectively. *K_L_* (L/g) and *a_L_* (L/mg) are the Langmuir isotherm constants. The constants *K_L_* and *a_L_* can be calculated from the intercept (1/*K_L_*) and the slope (*a_L_*/*K_L_*) of the linear plot of between *C_e_*/*q_e_* and *C_e_*. *q_max_* is the maximum adsorption capacity of the polymer and is defined by *K_L_*/*a_L_*.

The essential characteristic of this isotherm can be expressed in terms of a dimensionless constant (*R_L_*), which is called the separation factor and is given as follows (10) [27]:

(10) $$R_{L}=\frac{1}{1+a_{L}C_{o}}$$

where *C_o_* is the initial concentration of dye (mg/L) and *a_L_* is the Langmuir constant related to the energy of adsorption (L/mg). Depending on the value of *R_L_*, the adsorption process can be an unfavourable process (*R_L_* > 1), linear (*R_L_* = 1), favourable (*R_L_* between 0 and 1) or irreversible (*R_L_* = 0) [28].

The Temkin equation considered the effects of some indirect adsorbate/adsorbent interactions on adsorption isotherms and suggested the heat of adsorption of all molecules in the layer decreases linearly with coverage because of those interactions. The adsorption is characterized by a uniform distribution of bond energies until a bond energy maximum [29]. The linear form of Temkin relationship can be given as follows (11):

(11) $$q_{e}=\frac{RT}{b_{T}}\ln a_{T}+\frac{RT}{b_{T}}\ln C_{e}$$

where *T* is the absolute temperature in Kelvin, *R* is the universal gas constant (8.314 J/mol K), *a_T_* is the constant of Temkin isotherm (L/g) and *b_T_* is the Temkin constant, related to the heat of adsorption (kJ/mol). The Temkin constants *a_T_* and β (RT/ *b_T_*) are calculated from the slope and intercept of the linear plot of *q_e_* versus ln *C_e_*.

### 2.10. Thermodynamic Study

Thermodynamic consideration of an adsorption process is needed to conclude whether the process is exothermic or endothermic. Thermodynamic behavior is interpreted by the thermodynamic parameters, including Δ*H*° (standard enthalpy change), Δ*S*° (standard entropy change) and Δ*G*° (Gibbs free energy change). Thermodynamic parameters play an important role in determining heat change during the adsorption process and can be calculated by using the thermodynamic equilibrium coefficient obtained at different temperatures and concentrations. The Δ*G*° value is the essential criterion of spontaneity, and a negative value for Δ*G*° indicates the spontaneity of the reaction [30]. Assuming that the activity coefficients are the unit at low concentrations, the relationship of these thermodynamic parameters with adsorption equilibrium constant *K_c_* is given by the following classical Van’t Hoff Equation (12) [10]:

(12) $$K_{e}=1000\left(\frac{q_{e}}{C_{e}}\right),\;logK_{e}=\frac{\Delta S{}^{\circ}}{2.303RT}+\frac{-\Delta H{}^{\circ}}{2.303RT},\;\Delta G{}^{\circ}=\Delta H{}^{\circ}+T\Delta S{}^{\circ}$$

where *K*_e_ is the equilibrium constant, and *q_e_* and *C_e_* are the equilibrium concentrations of DB78 on polymers (mg/g) and in the solution (mg/L), respectively. *R* is the universal gas constant (8.314 J/mol K) and *T* is the absolute temperature in Kelvin. Δ*H*° and Δ*S*° parameters can be calculated from the slope and intercept of plot Log *K_e_* versus 1/*T*, respectively. 

### 2.11. Advanced Oxidation Process

The degradation of the dye by an AOP was conducted in a pulsed light device (XeMaticA-1L-Basic, Steribeam, Germany). A petri dish with 20 mL of a mixture of dye/H_2_O_2_ was treated with 60 flashes as previously reported [15]. Initial concentrations of dye were 14.4 and 49.1 mg/L, which corresponds to the remaining concentrations obtained after adsorbing a 300 mg/L dye solution by β-CDs-EPI and chitosan polymers respectively. The concentrations of H_2_O_2_ in the mixture were 200 times higher than those of the dye on the molar basis in order to keep an excess of H_2_O_2_ during the AOP. The decrease in absorbance of the mixture was monitored at 612 nm. Results are reported in terms of fluence (J/cm^2^), which is the amount of energy that impinges sample surface per unit of surface.

## 3. Results

### 3.1. Polymer Characterization

The characterization of both adsorbents involved the measure of the swelling capacity, porosity, density and the particle size distribution of each polymer. The results obtained could be observed in Table 1. It could be noted that the chitosan polymer has a swelling capacity greater than the β-CDs polymer, however, the β-CDs polymer presented a higher porosity. In the case of the particle size distribution, similar results were observed for both adsorbents.

The control of the adsorption process of an adsorbent, in this case, chitosan-NaOH and β-CDs-EPI polymers, depends on different physical-chemical characteristics. First, the type of polymer prepared, its chemical structure and functional groups and secondly the chemical characteristics of the molecule to be adsorbed, as well as its concentration and finally, the temperature.

### 3.2. Effect of Contact Time

**Figure 3:**
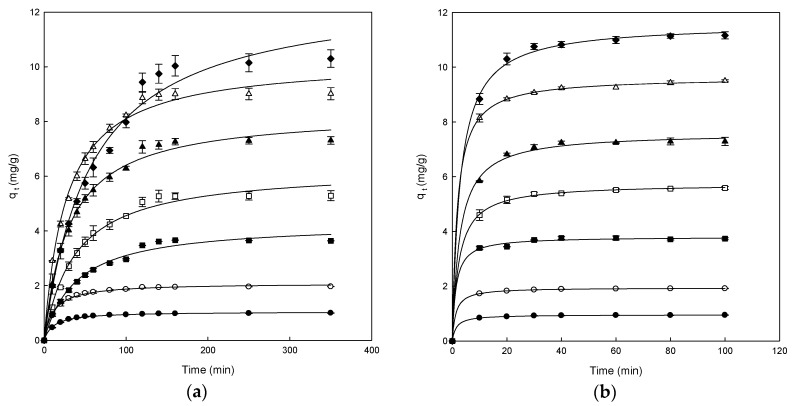
The effect of contact time between chitosan-NaOH (**a**) and β-CDs-EPI (**b**) at different concentrations of Direct Blue 78 25 mg/L (●), 50 mg/L (○), 100 mg/L (■), 150 mg/L (□), 200 mg/L (▲), 250 mg/L (Δ) and 300 mg/L (♦). The curves were adjusted using a hyperbolic mathematical model.

The adsorption data for the removal of DB78 versus contact time at different concentrations of dye (from 25 to 300 mg/L) for the polymers are shown in Figure 3. All the experiments were carried out at pH 7.0, constant stir (500 rpm) and fixed amount of polymer (1 g).

Figure 3a shows that the adsorption capacity increased in each concentration until the equilibrium was reached for the chitosan-NaOH polymer, indicating that the adsorption of dye on the polymer stopped. When this point is achieved, the amount of adsorbed dye inside the polymer was in a dynamic equilibrium with the amount of dye desorbed. The time needed to reach this point is called equilibrium time and the amount of dye removed by the polymer at that time indicates the maximum adsorption capacity of each polymer under these conditions.

Different adsorption phases may be differentiated in the range of concentrations analyzed (25–300 mg/L). In the case of adsorption of DB78 on the chitosan-NaOH polymer, at low concentrations, only 30 min were needed to reach the equilibrium. At the concentration of 100 mg/L, the adsorption was fast but it was slower than at low concentrations, reaching the equilibrium after 30 min of contact time. However, when the concentrations of dye were higher (>100 mg/L), the curves did not show the typical asymptotic form. Conversely, the equilibrium time increased with increasing concentrations of DB78 (from 150 to 300 mg/L).

For β-CDs-EPI polymers, the results obtained were different from those of the chitosan-NaOH polymers (Figure 3b). The adsorption was fast at the initial period between the dye and polymer, because of the fast linkage between the dye and the polymer surface. The adsorption is continuous until the equilibrium is reached after it remains constant. All curves were asymptotic after 20 min of contact time approximately. The adsorption process could be considered fast due to the high concentration of dye adsorbed in the initial period. When the concentration increased, the polymer incremented the ability to entrap the dye, confirming the strong interaction between DB78 and the β-CDs-EPI polymer.

### 3.3. Adsorption Kinetics

In order to test the experimental data obtained in the adsorption of DB78 and the β-CDs-EPI and chitosan polymers, the pseudo-first-order, pseudo-second-order and the intraparticle diffusion model were employed with the purpose to determine the different mechanisms implicated in the adsorption process, such as adsorption surface, mass transfer or intraparticle diffusion.

**Figure 4:**
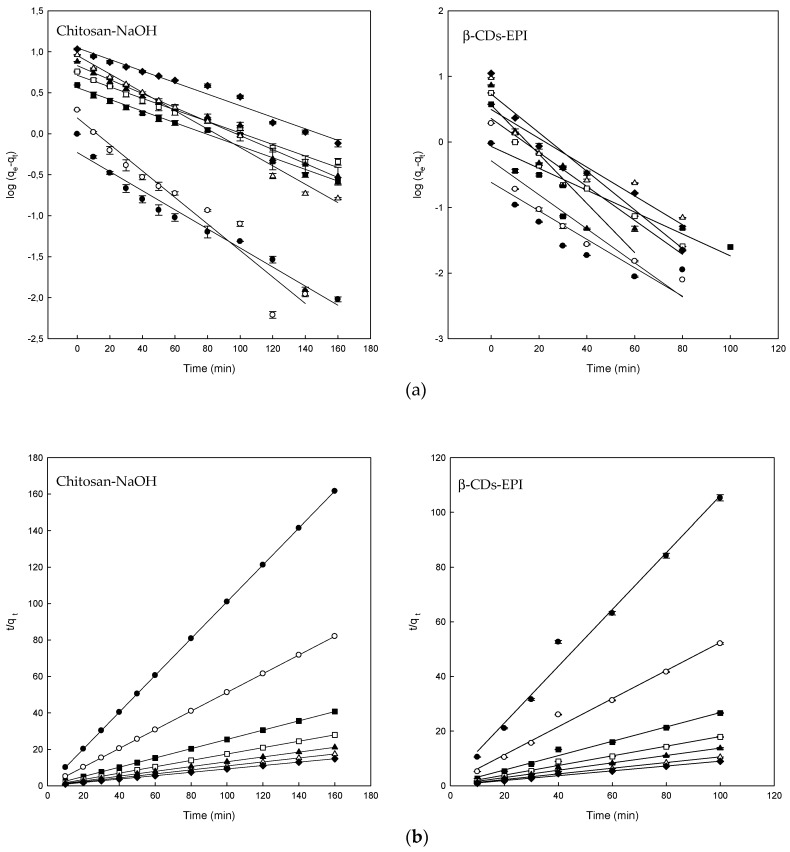
(**a**) The pseudo-first-order model plots and (**b**) pseudo-second-order model plots for the Direct Blue 78 adsorption onto chitosan-NaOH and β-CDs-EPI polymers at different concentrations of dye 25 mg/L (●), 50 mg/L (○), 100 mg/L (■), 150 mg/L (□), 200 mg/L (▲), 250 mg/L (Δ) and 300 mg/L (♦).

The fitting of experimental data to the pseudo-first-order plots for the adsorption of Direct Blue on different polymers are shown in Figure 4a and the corresponding parameters in Table 2. The goodness of fit of this model was expressed using the linear determination coefficient (R^2^). The linearity of the model (log (*q_e_* − *q_t_*) versus *t*) was plotted for 350 min of contact in the case of the chitosan-NaOH polymer and 100 min for the β-CDs-EPI polymer. The R^2^ values for chitosan-NaOH ranged from 0.934 to 0.986 and between 0.736 and 0.938 for β-CDs-EPI. Theoretical values of *q_e_* were compared with the experimental data. Despite the fact that some values were relatively high, the obtained R^2^ value revealed the poor fit to the model. Because of these results, it was appropriate to study the pseudo-second-order model with the experimental data obtained.

The plot of *t*/*q_t_* versus *t* produced straight lines in all cases during the whole range of measure, as observed in Figure 4b. In this model, a relatively high value indicates also that the experimental data fit properly to the model. R^2^ and *q_e_* values obtained indicated that better results were obtained with this model, as it is shown in Table 2. In all cases, the R^2^ values were 0.99 or higher and the experimental *q_e_* fitted much better to theoretical values of *q_e_* than the pseudo-first-order model. These results suggested that the adsorption process of DB78 is controlled by the pseudo-second-order model and supports that the adsorption is due to chemisorption or chemical adsorption. The adsorption is carried out by surface exchange reactions, DB78 molecules diffuse inside the polymer where inclusion complexes, hydrogen bonds or hydrophobic interactions could take place [22]. Similar kinetics were also observed in the adsorption of Direct Red dye using CDs-EPI polymers [16,28] or adsorption of three different dyes using chitosan [31]. 

The adsorption is a process with different stages that implicates a transport of solute molecules (dye) from the aqueous solution to the surface of solid particles (polymer), followed by the diffusion of dye molecules onto the polymer. The experiments developed, permitted us to study if the intraparticle diffusion is the process that controls the adsorption. This effect was studied by plotting the amount of dye adsorbed versus the square root of time and this is the intraparticle diffusion model. The kinetics results obtained can be used to know if the intraparticle diffusion is the limiting step in the adsorption of the dye inside the polymer [21,22].

**Figure 5:**
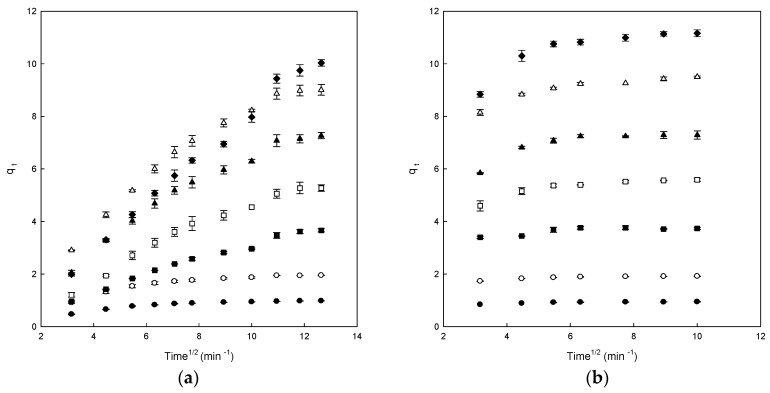
The intraparticle diffusion model plots for the Direct Blue 78 adsorption onto chitosan-NaOH (**a**) and β-CDs-EPI (**b**) polymers at different dye concentrations (25 mg/L (●), 50 mg/L (○), 100 mg/L (■), 150 mg/L (□), 200 mg/L (▲), 250 mg/L (Δ) and 300 mg/L (♦)).

Figure 5 shows the amount of adsorbed dye versus the square root of time for the intraparticle transport of DB78 for both studied polymers and different concentrations of dye. The curves presented multi-steps. In the case of β-CDs-EPI (Figure 5b), the curves presented two zones, the first curved part indicated the effect of the boundary layer and the second linear part is due to the intraparticle diffusion.

However, in the case of a chitosan-NaOH polymer, a single linear portion for all studied concentration was observed, indicating that the adsorption process inside the polymer was controlled by diffusion of such molecules to the polymer surface (Figure 5a). 

The *k_i_* (intraparticle diffusion constant) values could be seen in Table 2. These values increased with increasing concentrations of DB78 in both polymers. Chitosan-NaOH presented better R^2^ values than β-CDs-EPI, ranging from 0.627 to 0.814 for β-CDs-EPI and from 0.804 to 0.991 for chitosan-NaOH.

However, the results obtained for each curve do not cross the origin, indicating that the intraparticle diffusion is not the sole rate-limiting step although it plays an important role in the adsorption process, other process control adsorption velocity, confirming that the adsorption is a process that involves different stages [22]. 

The C (*q_e_*) values obtained provide information about the thick of the boundary layer; a higher intercept indicates a higher effect of it. The C (*q_e_*) values increased with increasing dye concentrations for β-CDs-EPI polymer but not for chitosan-NaOH (Table 2) [19], therefore, there is intraparticle diffusion for the β-CDs-EPI polymer but not for the chitosan-NaOH polymer.

Similar kinetics were also observed in the adsorption of Direct Red dye using CDs-EPI polymers [16,28]. As it may be observed in this study, the intraparticle diffusion is not the unique mechanism implicated in the process, though it plays an important role in the adsorption process, as with the adsorption of DB78 with a β-CDs-EPI polymer. However, the results are clearly different from that obtained with the chitosan-NaOH polymer.

### 3.4. Adsorption Equilibrium

**Figure 6:**
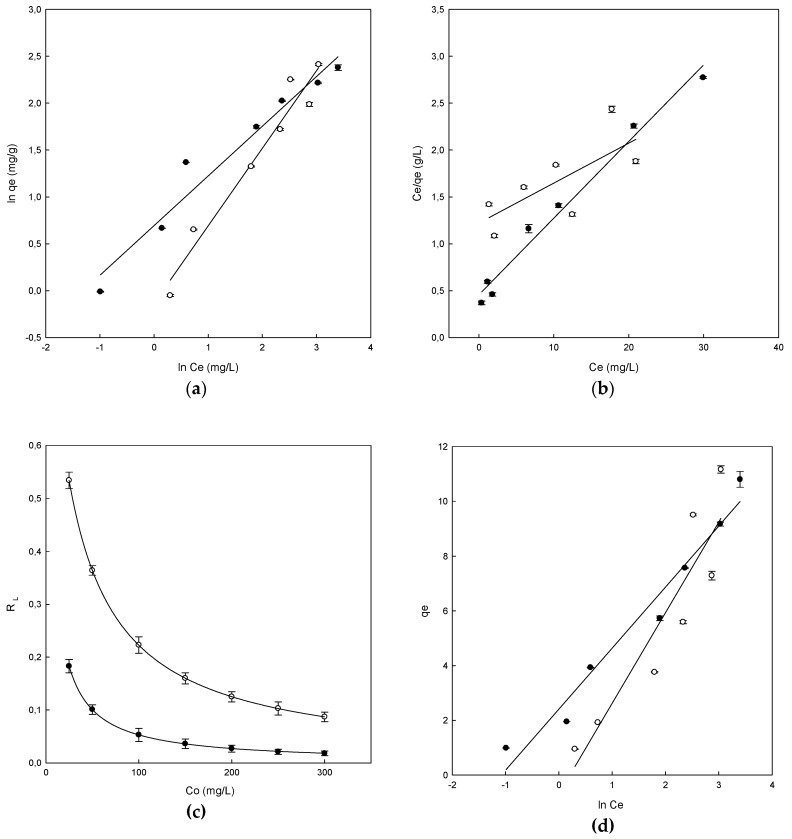
The adsorption isotherms for Direct Blue 78 by chitosan-NaOH (●) and β-CDs-EPI (○). (**a**) Freundlich isotherm, (**b**) Langmuir isotherm, (**c**) Separation factor, (**d**) Temkin isotherm.

The experimental data of adsorption equilibrium obtained with DB78 and CDs-EPI polymers and chitosan were studied using Freundlich, Langmuir and Temkin isotherms. The representation of the Freundlich isotherm was obtained by the plot of ln *q_e_* versus ln *C_e_*. The linear plot was obtained for both polymers as may be observed in Figure 6a. These straights lines were used to calculate the parameters *K_F_*, *n_F_* and R^2^. The value of the Freundlich constant (*K_F_*) was 0.87 (L/g) for β-CDs-EPI, while this value was 2.0 (L/g) for chitosan-NaOH (Table 3). Using the linear plot, the value of Freundlich exponent (*n_F_*) could also be calculated, this value was 1.2 for β-CDs-EPI and 1.88 for chitosan-NaOH (Table 3). The adsorption process is favorable when the value of *n_F_* is in the range between 1–10 and this is confirmed for both polymers. The values of R^2^ obtained were 0.954 for β-CDs-EPI and 0.956 for chitosan-NaOH (Table 3). It was found that the adsorption equilibrium data fit the Freundlich equation due to the high determination coefficient obtained.

The plot of *C_e_*/*q_e_* versus *C_e_* shows the representation of the Langmuir isotherm, giving a straight line in both polymers, whose slope is *a_L_*/*K_L_*, the intercept is 1/*K_L_* and *K_L_*/*a_L_* is the parameter *q_max_* which is the maximum adsorption capacity of each polymer (mg/g) (Figure 6b).

The parameters obtained for this isotherm can be observed in Table 3. One of the most useful parameters of this model is *q_max_*. The value obtained for β-CDs-EPI was 23.47 and 12.30 mg/g for chitosan-NaOH, indicating that β-CDs-EPI presented a better adsorption capacity of Direct Blue than chitosan-NaOH. In our previous research [16], a table comparing the q_max_ of different adsorbents and dyes was included (see Table 4). The results observed in this article are consistent with those previously compared.

Furthermore, the R^2^ values obtained were 0.516 for β-CDs-EPI and 0.981 for chitosan-NaOH. This value was lower to that obtained with the Freundlich isotherm for β-CDs-EPI, however, it was higher to that obtained with the Freundlich isotherm for chitosan-NaOH. Similar results were also observed in the adsorption of Basic Blue 9 dye using CDs polymers [25], and Pellicer et al. in the adsorption of Direct Red dye using CDs-EPI polymers [16,28]. In both cases, the adsorption process fitted to the Langmuir isotherm. On the other hand, studies carried out by Subramani and Thinakaran showed similar results for the chitosan-NaOH polymer in the adsorption of three different dyes [31].

In the Langmuir isotherm, to know if the adsorption process is considered favorable or unfavorable, the value of a dimensionless constant called separation factor (*R_L_*) is taken into account. When the value of *R_L_* is in the range between 0–1, it indicates that the process is favorable and this is observed by the values obtained between 0–1 in both polymers, as observed in Figure 6c. It is also observed that the highest values of *R_L_* at low concentrations of dye indicate that the adsorption is more favorable at low concentrations. 

The experimental data were also analyzed using the Temkin isotherm to study its fit. The parameters calculated with the Temkin isotherm as well as the values of determination coefficients could be observed in Table 3. The equation of the Temkin isotherm assumes that because of the interactions between polymer and dye, the heat of adsorption of all the molecules in the layer would decrease linearly with coverage of the adsorbent surface, as well as that the adsorption is characterized by uniform distribution of bond energies, until a bond energy maximum [29]. From the plot of *q_e_* versus ln *C_e_* (Figure 6d), the linear form of the isotherm was plotted as well as the parameters *b_T_* and *a_T_* using the slope and the intercept respectively.

The *b_T_* value obtained for the different polymers were 0.733 kJ/mol for β-CDs-EPI and 1.09 kJ/mol for the chitosan-NaOH polymer. Positive values indicated that in the adsorption process were involved physic as well as chemisorption processes. The values of R^2^ obtained were 0.844 for β-CDs-EPI and 0.965 for chitosan-NaOH.

### 3.5. Thermodynamic Study

**Figure 7:**
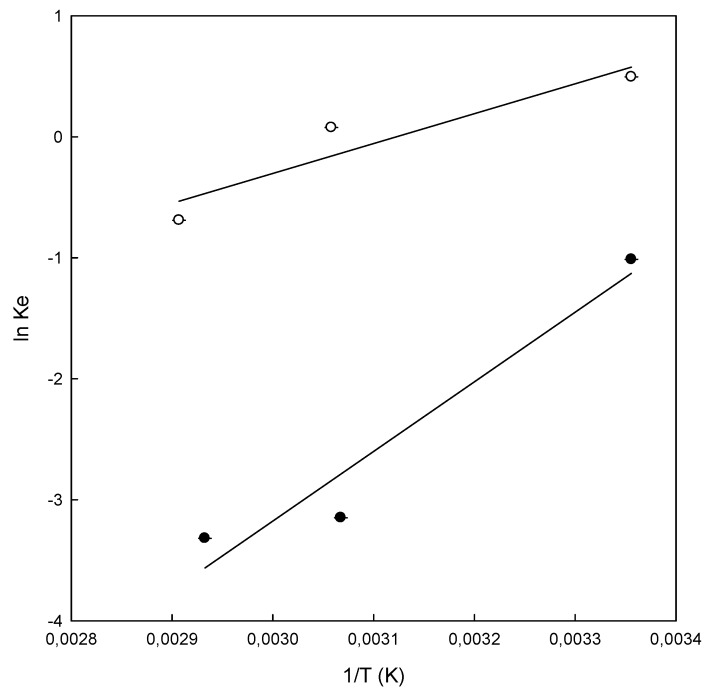
Van’t Hoff plot for the adsorption of Direct Blue 78 onto chitosan-NaOH (●) and β-CDs-EPI (○) polymers at different temperatures. K_e_ is the equilibrium constant and T the temperature.

With the objective to study the effect of temperature on the adsorption of DB78 by chitosan-NaOH and β-CDs-EPI polymers, the experiments were carried out at three different temperatures (Figure 7) at a concentration of 250 mg/L for both polymers. Table 4 presents the values of thermodynamic parameters obtained for both polymers at different temperatures.

The standard free energy (ΔG°) of the adsorption of DB78 for β-CDs-EPI, calculated using Van’t Hoff equation, was −1.22, −0.21 and 1.97 kJ/mol at temperatures of 25, 54 and 71 °C. In the case of a chitosan-NaOH polymer, the values obtained were 2.5, 8.5 and 9.4 kJ/mol at temperatures of 25, 53 and 68 °C respectively. The negative values of ΔG° for β-CDs-EPI polymer confirm the viability of the process and the spontaneous nature of the adsorption process at 25 and 54 °C, but it is not spontaneous at higher temperatures (71 °C). On the other hand, positive values of ΔG° for chitosan-NaOH polymer indicated that the process is not spontaneous at all temperatures studied. The enthalpy change (ΔH°) was negative in both polymers, indicating the exothermic nature of the adsorption process for the dye. Similar results were observed by Subramani and Thinakaran [31] in the adsorption of three different dyes using chitosan. The results showed that it was an exothermic process. In relation to the standard free energy, the process was spontaneous, in contrast to the ΔG° obtained with DB78. Table 4 shows that the value of ΔG° increased with increasing temperatures in both polymers. In the case of chitosan-NaOH, the value increased from 2.50 to 9.40 kJ/mol and from −1.22 to 1.97 kJ/mol for β-CDs-EPI, which indicates a clear trend in the process. High temperatures were not suitable for the adsorption process, however, this process was favored at low temperatures.

**Figure 8:**
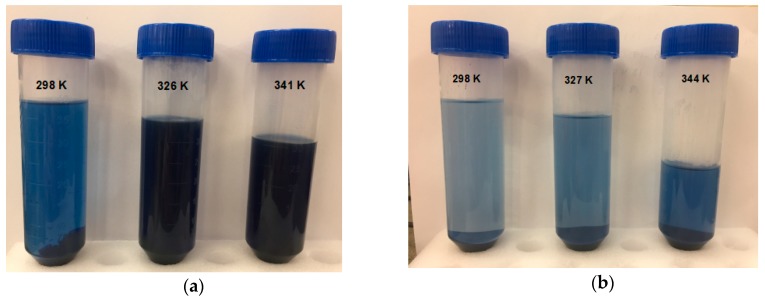
The effect of temperature for the adsorption of Direct Blue 78 onto chitosan-NaOH (**a**) and β-CDs-EPI (**b**) polymers at a concentration of 250 mg/L at different temperatures.

Figure 7 shows the results obtained in the thermodynamic study. As stated before, at temperatures of 53 and 68 °C, there was not an increase in the adsorption properties of chitosan-NaOH (Figure 8a). In the case of the β-CDs-EPI polymer, the best conditions to adsorb the dye was achieved at 25 °C, decreasing the capability of this polymer to entrap more dye molecules after increasing the temperature (Figure 8b).

### 3.6. Advanced Oxidation Process

**Figure 9:**
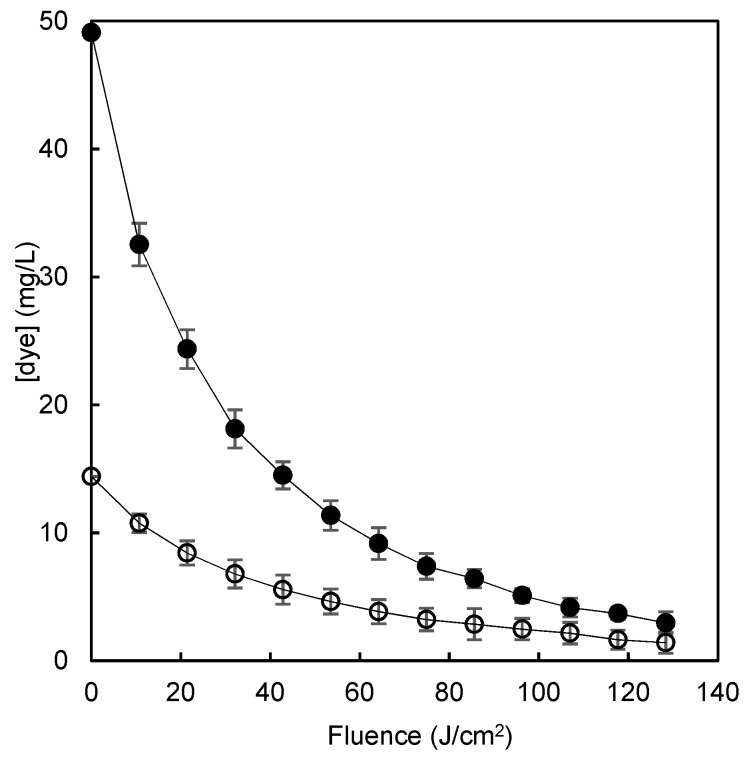
The degradation of Direct blue 78 by a pulsed light/H_2_O_2_ advanced oxidation process in model wastewater after chitosan-NaOH (o) or β-CDs-EPI (●) polymer adsorption.

The highest residual concentrations of dye remaining after adsorption by each polymer were chosen as the worst case scenario for further improvement of the process with the aim of minimizing the amount of dye that eventually would be disposed to the environment. The adsorption of 300 mg/L of the dye solution by chitosan polymer or β-CDs-EPI polymer retained 83.6% and 95.2% of the dye respectively, leaving a residual concentration of dye of 49.1 and 14.4 mg/L respectively. The application of the AOP to the solutions remaining after the adsorption processes reduced their concentration by more than 90% (Figure 9) in both cases and rose the dye removal rate of the sequential polymer adsorption/AOP system to >99%.

## 4. Conclusions

Chitosan-NaOH and β-CDs-EPI polymers can be successfully used in the removing of DB78. The process of adsorption in both polymers was found to be better described by the pseudo-second-order kinetic model with a good determination coefficient, which supports that it is a chemisorption process. The adsorption equilibrium of DB78 onto chitosan-NaOH was suitably described by the Langmuir model and onto β-CDs-EPI was described by the Freundlich isotherm, being the maximum adsorption capacity of 12.30 and 23.47 mg/g for chitosan-NaOH and β-CDs-EPI, respectively. The thermodynamic analysis indicates that the process of adsorption is exothermic in both cases and spontaneous at lower temperatures for β-CDs-EPI. The complementary action of an innovative advanced oxidation process brought about a wastewater with less than 1% of the initial dye concentration. This study demonstrated that the chitosan-NaOH and β-CDs-EPI polymers can be an effective and eco-friendly adsorbent material for the removal of DB78 dye from colored wastewater.

## Figures and Tables

**Table 1:** The swelling capacity, porosity and particle size distribution of both adsorbents.

Polymers Properties	Chitosan-NaOH Polymer	β-CDs-EPI Polymer
**Swelling capacity (Q_eg_, *g*/*g*)**	0.80 ± 0.01	0.63 ± 0.01
**Porosity (%)**	37.6 ± 0.3	54.1 ± 1.1
**Density (g/cm^3^)**	1.4 ± 0.1	1.5 ± 0.2
**Particle size distribution D_[4:3]_ (µm)**	350	586

**Table 2:** The kinetics parameters of the Pseudo-First, Pseudo-Second-Order and Intraparticle Diffusion Models for the adsorption of Direct Blue 78 onto chitosan-NaOH and β-CDs-EPI polymers.

**PFOM ^1^**	**Chitosan-NaOH**					**β-CDs-EPI**		
**Co (mg/L)**	**q_eexp_**	**q_ecal_**	**k_1_ (min^−1^)**	**R^2^**	**q_eexp_**	**q_ecal_**	**k_1_ (min^−1^)**	**R^2^**
**25**	0.990	0.590	0.026	0.971	0.950	0.242	0.049	0.751
**50**	1.950	1.560	0.037	0.934	1.920	0.517	0.059	0.950
**100**	3.930	3.640	0.016	0.980	3.760	0.850	0.038	0.736
**150**	5.730	5.150	0.016	0.986	5.590	2.280	0.059	0.919
**200**	7.570	6.780	0.020	0.983	7.290	3.650	0.086	0.890
**250**	9.170	9.000	0.026	0.954	9.500	3.120	0.050	0.839
**300**	10.800	11.140	0.016	0.979	11.160	5.370	0.067	0.938
**PSOM ^2^**	**Chitosan-NaOH**					**β-CDs-EPI**		
**Co (mg/L)**	**q_eexp_**	**q_ecal_**	**K_2_ (min^−1^)**	**R^2^**	**q_eexp_**	**q_ecal_**	**K_2_ (min^−1^)**	**R^2^**
**25**	0.990	1.000	0.045	0.999	0.950	0.950	0.0135	0.999
**50**	1.950	1.950	0.093	0.999	1.920	1.920	1.920	0.999
**100**	3.930	3.930	0.285	0.999	3.760	3.730	3.730	0.999
**150**	5.730	5.730	0.430	0.999	5.590	5.620	5.620	0.999
**200**	7.570	7.570	0.534	0.999	7.290	7.300	7.300	0.999
**250**	9.170	9.160	0.644	0.999	9.500	9.520	9.520	0.999
**300**	10.800	10.790	0.864	0.999	11.160	11.240	11.240	0.999
**IDM ^3^**	**Chitosan-NaOH**					**β-CDs-EPI**		
**Co (mg/L)**	**q_eexp_**	**q_ecal_**	**K_i_ (mg/g min^1/2^)**	**R^2^**	**q_eexp_**	**q_ecal_**	**K_i_ (mg/g min^1/2^)**	**R^2^**
**25**	0.990	0.475	0.045	0.804	0.950	0.827	0.0135	0.750
**50**	1.950	0.930	0.093	0.806	1.920	1.700	0.0241	0.771
**100**	3.930	0.233	0.285	0.981	3.760	3.315	0.0495	0.627
**150**	5.730	0.260	0.430	0.962	5.590	4.495	0.124	0.761
**200**	7.570	1.000	0.534	0.959	7.290	5.850	0.170	0.633
**250**	9.170	1.610	0.644	0.952	9.500	7.940	0.170	0.814
**300**	10.800	−0.522	0.864	0.991	11.160	8.732	0.279	0.699

^1^ PFOM: Pseudo-first-order model; ^2^ PSOM: Pseudo-second-order model; ^3^ IDM: Intraparticle diffusion model.

**Table 3:** The adsorption isotherm constants obtained for the chitosan-NaOH and β-CDs-EPI polymers.

Isotherm	Parameter	Chitosan-Naoh	β-CDS-EPI
**Freundlich**	K_F_	2.0	0.87
	n_F_	1.88	1.2
	R^2^	0.956	0.954
**Langmuir**	q_max_	12.30	23.47
	K_L_	2.187	0.819
	a_L_	0.178	0.035
	R^2^	0.981	0.516
	R_L_	0.183–0.018	0.534–0.087
**Temkin**	a_T_	2.94	0.81
	b_T_	1.09	0.733
	R^2^	0.965	0.844

**Table 4:** Thermodynamic parameters for the adsorption of Direct Blue 78 onto chitosan-NaOH and β-CDs-EPI polymers at different temperatures.

Polymers	T (K)	ΔG°(kJ/mol)	ΔH° (kJ/mol)	ΔS° (kJ/K·mol)
**Chitosan-NaOH**	298	2.50	−47.85	−0.17
	326	8.50	-	-
	341	9.40	-	-
**β-CDS-EPI**	298	−1.22	−20.52	−0.06
	327	−0.21	-	-
	344	1.97	-	-
